# Clinical value of BISAP score combined with low-density lipoprotein cholesterol and ascites in early prediction of hypertriglyceridemic severe acute pancreatitis

**DOI:** 10.3389/fendo.2025.1722057

**Published:** 2025-12-09

**Authors:** Xiaofen Xu, Tao Xu, Ye Wang, Lei Li

**Affiliations:** Department of Gastroenterology, Beijing Jishuitan Hospital, Capital Medical University, Beijing, China

**Keywords:** hypertriglyceridemic acute pancreatitis, low-density lipoprotein cholesterol, BISAP score, ascites, severity

## Abstract

**Background and aims:**

An immediate diagnosis and accurate severity assessment of hypertriglyceridemic acute pancreatitis (HTG-AP) are essential. However, risk scoring system specific to HTG-AP has not yet been established. The study aims to identify the potential early predictors of severe cases and explore a novel predictive model for its early identification.

**Methods:**

A retrospective analysis was conducted on 189 patients with HTG-AP. According to the revised Atlanta classification, patients were categorized into severe (HTG-SAP, N = 25) and no severe (HTG-NSAP, N = 164) groups. Independent predictors of HTG-SAP were identified through univariate and multivariate logistic regression analyses, with predictive abilities estimated by receiver operating characteristic curves.

**Results:**

Univariate analysis revealed statistically significant differences in several parameters, including diabetes mellitus prevalence, the BISAP score, serum biomarkers, such as low-density lipoprotein cholesterol (LDL-C), and the ascites incidence, between the two groups (*P* < 0.05). According to multivariate logistic regression, the BISAP score, low LDL-C level, and the presence of ascites were identified as independent risk factors for HTG-SAP, with the area under the curve (AUC) values of 0.840, 0.702, and 0.742, respectively. The AUC for the model combined BISAP with LDL-C and ascites was 0.900. Decision curve analysis demonstrated that implementing clinical interventions for cases identified by the model as being at risk of developing HTG-SAP could yield significant clinical benefits.

**Conclusions:**

The BISAP score combined with LDL-C and ascites might have a potential early predictive value for HTG-SAP, and additional multicenter studies involving larger samples are needed to confirm these results.

## 1 Introduction

Acute pancreatitis (AP) represents a critical gastroenterological emergency necessitating prompt diagnosis and urgent therapeutic intervention, characterized by pancreatic inflammation and/or necrosis, which may lead to systemic inflammatory response syndrome (SIRS) and multiple organ dysfunction syndrome (MODS) (1, 2). Majority of AP patients have a self-limited and mild course with an overall mortality rate of about 5%, while approximately 15%-25% of patients may progress from moderately severe to severe acute pancreatitis (SAP), of which the mortality remains as high as 20-40% (2–4). AP has multiple etiologies, with the three major causes being gallstones, alcohol consumption, and hypertriglyceridemia (HTG). The incidence of hypertriglyceridemic acute pancreatitis (HTG-AP) has been increasing annually in Chine, surpassing alcohol to become the second cause of AP, due to the improvement in living standards and changes in dietary habits (5, 6). Recent research indicates that the prevalence of HTG-AP increased approximately 2.6-fold between 2012 and 2021 within the Chinese population (7). Meanwhile, in contrast to AP of other etiologies, HTG-AP is becoming more prevalent among younger individuals, and has worse clinical outcomes and higher recurrence rates (5, 8, 9). Recent studies showed that HTG-AP is associated with a substantially elevated risk of systemic complications, such as SIRS and organ failure (OF), as well as local complications (5, 10–12). These patients exhibit a heightened risk of progression to hypertriglyceridemic severe acute pancreatitis (HTG-SAP), with reported incidence rates ranging from 12% to 33% (6, 7, 13–16). Therefore, early and accurate severity assessment for HTG-AP assumes critical importance, particularly within the first 24 hours of hospitalization, thereby implementing proactive preventive measures and improving the prognosis.

It is well known that the clinical assessment of AP severity relies on multiple validated risk prediction scoring systems, including the Ranson score (17), Acute Physiology and Chronic Health Evaluation II (APACHE II) (18), the bedside index of severity in AP (BISAP) (19), the computed tomography (CT) severity index (CTSI) (20), and the modified CTSI (MCTSI) (21). However, most existing scoring systems are complex to operate and depend on longitudinal data collection. APACHE II score, for instance, incorporates numerous parameters that complicate calculation procedures. Ranson score requires 48-hour observation periods to generate results, while CTSI or MCTSI is typically applicable only after 48–72 hours post-admission. These limitations consequently restrict their utility in early-stage severity stratification in AP patients. The BISAP score is one of the most commonly utilized tools for early prediction of sever cases and mortality, offering the advantages of operational simplicity, rapid assessability, and feasibility for completion during the initial 24-hour period after hospital admission. Furthermore, some studies have shown that the predictive value of the existing scoring systems may be limited by the heterogeneous etiology of AP, and the BISAP score may be the best criterion in predicting severity and prognosis of HTG-AP (22, 23). However, previous studies have showed the BISAP score had poor sensitivity (24–26), and lacked incorporation of early laboratory parameters and abdominal imaging indicators, which limited its utility as a standalone tool. Evidence indicates that ascites is more prevalent in SAP and frequently develops as a mild to moderate effusion in the early stages. The presence of ascites is linked to an increased risk of OF, infected pancreatic necrosis (IPN), and mortality (27). Currently, a number of biomarkers have been used to predict the severity of HTG-AP at an early stage, such as C-reactive protein (CRP), lactate dehydrogenase (LDH), serum calcium (Ca^2+^), Neutrophil-Lymphocyte Ratio (NLR), MicroRNA-155, serum macrophage migration inhibition factor, and the orointestinal microbiome (14, 28–30). Although HTG is a known etiology of AP, its role in predicting disease severity is debated, with the majority of studies reporting no significant association (31, 32). Emerging evidence suggests that total cholesterol (TCHOL) and cholesterol-related lipids, such as low-density lipoprotein cholesterol (LDL-C), may be implicated in the pathogenesis and progression to severe disease in AP (33); however, this relationship warrants further investigation. Previous studies (29, 34–36) have indicated potential clinical correlations between combined scoring systems and blood-based biomarkers. The study aims to identify potential parameters demonstrating optimal accuracy for early prediction of HTG-AP severity, and to evaluate whether integrating these parameters with the BISAP score improves predictive capability.

## 2 Methods

### 2.1 Study design

This study conducted a retrospective analysis of clinical data from patients with HTG-AP admitted to Beijing Jishuitan Hospital, a tertiary hospital in China. This study was carried out in accordance with the principles of the Declaration of Helsinki and was approved by the ethics committee of Beijing Jishuitan Hospital, Capital Medical University (Approval No. K2025-022-00). The requirement for informed consent was waived.

### 2.2 Patients

From January 2021 to January 2025, we retrospectively analyzed 189 HTG-AP cases who met the inclusion and exclusion criteria. Inclusion criteria were as follows: 1) met the diagnostic criteria of AP according to the 2012 revised Atlanta classification (37); 2) serum triglyceride (TG) level reaching 11.3 mmol/L, or TG level reaching 5.65 mmol/L with chylous serum; 3) laboratory tests and CT of the abdomen and chest completed within 24 h after admission. Patients who met the following criteria were excluded: 1) other etiologies of AP, such as biliary, alcoholic, drug-induced, and endoscopic or surgical interventions; 2) acute onset of chronic pancreatitis; 3) pancreatic carcinoma; 4) more than 48 hours from symptom onset to hospital admission; 5) suspected or confirmed infectious diseases at other sites; 6) comorbidities including cirrhosis, coronary heart disease, chronic obstructive pulmonary disease, and chronic renal failure, among other acute and chronic conditions; 7) pregnancy; and 8) incomplete clinical data.

### 2.3 Severity classification

Patients were classified as mild (MAP; n = 54), moderately severe (MSAP; n = 110), and severe (SAP; n = 25) according to the 2012 revised Atlanta classification. MAP was characterized by the absence of both OF and local complications; MSAP was defined by transient OF (resolving within 48 hours) and/or local complications; and SAP was diagnosed in cases of persistent OF exceeding 48 hours. OF (encompassing respiratory, renal, and cardiovascular systems) was identified using the modified Marshall scoring system, with a score of ≥ 2 in any system constituting failure. Subsequently, the patients were divided into the HTG-SAP (N = 25) and hypertriglyceridemic non-severe acute pancreatitis (HTG-NSAP; N = 164) groups.

### 2.4 Data collection

A detailed history and physical examinations of all enrolled patients were conducted after admission. The general data of the two groups, including age, sex, body mass index (BMI), history of ordinary alcohol consumption, history of smoking, comorbidities (hypertension, diabetes mellitus and fatty liver), vital signs, presence or absence of psycho-behavioural or mental abnormalities, and hospitalisation time were meticulously recorded. Laboratory tests collected within 24 h of admission included white blood cell count (WBC), neutrophil count (NEU), red blood cell count (RBC), platelet count (PLT), haemoglobin (Hb), haematocrit (HCT), CRP, serum amylase (SAMY), blood gas analyses, TG, TCHOL, high-density lipoprotein cholesterol (HDL-C), LDL-C, alanine aminotransferase (ALT), aspartate aminotransferase (AST), alkaline phosphatase (ALP), total bilirubin (TBIL), gamma-glutamyl transferase (GGT), LDH, albumin, serum creatinine (Cr), blood urea nitrogen (BUN), blood glucose, blood Ca^2+^, and D-dimer (D-D). The peak values of all laboratory parameters and vital signs recorded during the observation period were selected for analysis. CT of the abdomen and chest was performed in all patients at 24 hours after admission. CT images of all patients were re-evaluated to document the presence or absence of pleural effusion and ascites. Subsequently, BISAP scores in the first 24 hours were recorded.

### 2.5 Statistical analysis

Statistical analyses were conducted with SPSS Statistics v.26.0 (IBM Corp, Armonk, NY, USA) and R 4.1.2 software. Continuous variables were tested for normal distribution by the Kolmogorov–Smirnov test. Those with a normal distribution were expressed as means (SD, standard deviations) and compared by the independent sample t-test, while continuous variables with a skewed distribution were presented as medians (IQR, interquartile ranges) and analyzed by the Mann-Whitney U-test. Categorical variables were expressed as frequency or proportion, and analyzed by Pearson’s chi-square test or Fisher’s exact test. Two-sided *P* < 0.05 was considered statistically significant. In univariate analysis, parameters showing significant differences (*P* < 0.05) were selected. The candidate predictors were then incorporated into a multivariate logistic regression model (enter method) to identify independent predictors of HTG-SAP. These were then used to build the final predictive model. Receiver operating characteristic (ROC) curves were constructed, and the area under the curve (AUC) along with optimal cutoff values were determined to quantify the predictive performance of each independent predictive variable and the predictive model. Sensitivity, specificity, and the maximum Youden index were concurrently calculated. A decision curve analysis (DCA) plot was constructed for the novel predictive model to evaluate its clinical utility. Finally, the Bootstrap method was used to repeat the sampling 1000 times for internal validation.

## 3 Results

### 3.1 General characteristics of the HTG-NSAP and HTG-SAP groups

In total, 189 patients with HTG-AP were enrolled in this study, including 164 cases (86.8%) in the HTG-NSAP group and 25 cases (13.2%) in the HTG-SAP group. **Table 1** showed the general characteristics of the cohort. There were no significant differences in age, sex, BMI, regular drinking and smoking history, comorbidities of hypertension and fatty liver between the two groups (all *P* > 0.05). The proportion of diabetes mellitus in the HTG-SAP group was higher than that in the HTG-NSAP group (*P* < 0.05). The HTG-SAP group had prolonged length of hospital stay compared with the HTG-NSAP group (*P* < 0.05).

**Table 1:** General characteristics of the HTG-NSAP and HTG-SAP groups.

Variables	HTG-NSAP group (N = 164)	HTG-SAP group (N = 25)	*χ*^2^/*Z*	*P* value
Age, years	37.0 (33.0, 44.0)	37.0 (31.3, 43.3)	*Z*=-0.617	0.537
Sex, n (%)			*χ*^2^ = 0.139	0.709
Male	130 (79.3)	19 (76.0)		
Female	34 (20.7)	6 (24.0)		
BMI, kg/m^2^	27.68 (25.71, 30.41)	29.15 (26.60, 33.65)	*Z*=-1.698	0.090
Smoking, n (%)	86 (52.4)	11 (44.0)	*χ*^2^ = 0.618	0.432
Normal drinking, n (%)	59 (36.0)	8 (32.0)	*χ*^2^ = 0.150	0.699
Hypertension, n (%)	36 (22.0)	7 (28.0)	*χ*^2^ = 0.452	0.502
Diabetes mellitus, n (%)	75 (45.7)	18 (72.0)	*χ*^2^ = 5.989	0.014
Fatty liver, n (%)	135 (82.3)	24 (96.0)	*χ*^2^ = 2.103	0.147
Hospital stays, days	8.0 (6.0, 10.0)	12.0 (10.0, 16.0)	*Z*=-5.231	0.000

HTG-NSAP, hypertriglyceridemic non-severe acute pancreatitis; HTG-SAP, hypertriglyceridemic severe acute pancreatitis; BMI, body mass index.

### 3.2 Clinical parameters of the HTG-NSAP and HTG-SAP groups

There were no significant differences in HCT, RBC, PLT, Hb, TG, ALT, AST, ALP, TBIL, GGT, albumin, Cr, and BUN between the two groups (*P* > 0.05). Compared with the HTG-NSAP group, the HTG-SAP group presented significantly higher levels of WBC, NEU, CRP, SAMY, TCHOL, LDH, glucose, and D-D (*P* < 0.05). Furthermore, the value of BISAP score, as well as the incidence of ascites were significantly higher than those in HTG-NSAP group (*P* < 0.05). Conversely, the HTG-SAP group demonstrated significantly reduced levels of HDL-C, LDL-C, and Ca^2+^ relative to the HTG-NSAP group (*P* < 0.05) (**Table 2**).

**Table 2:** Clinical parameters of the HTG-NSAP and HTG-SAP groups.

Variables	HTG-NSAP group (N = 164)	HTG-SAP group (N = 25)	*χ*^2^/*Z*	*P* value
WBC, ×10^9^/L	12.94 (10.28, 15.54)	15.43 (12.53, 21.39)	*Z*=-3.004	0.003
NEU, ×10^9^/L	10.26 (7.07, 12.75)	12.03 (9.26, 16.37)	*Z*=-2.598	0.009
HCT, L/L	43.50 (40.40, 46.10)	42.80 (39.90, 44.57)	*Z*=-0.973	0.330
RBC (×10^12^/L)	5.16 (4.66, 5.50)	5.06 (4.69, 5.32)	*Z*=-0.514	0.607
PLT (×10^9^/L)	275.50 (226.00, 323.00)	268.00 (230.25, 331.50)	*Z*=-0.271	0.787
Hb, g/L	159.00 (144.00, 168.00)	158.00 (145.25, 170.75)	*Z*=-0.351	0.725
CRP, mg/L	20.08 (3.56, 89.57)	47.34 (6.74, 163.17)	*Z*=-1.994	0.046
SAMY, U/L	194.00 (85.00, 408.00)	325.00 (202.00, 734.00)	*Z*=-2.231	0.026
TG, mmol/L	19.66 (9.71, 31.54)	25.81 (5.51, 37.40)	*Z*=-0.981	0.327
TCHOL, mmol/L	8.89 (6.64, 12.14)	13.14 (7.38, 17.70)	*Z*=-2.763	0.006
HDL-C, mmol/L	0.88 (0.75, 1.03)	0.74 (0.66, 0.82)	*Z*=-3.784	0.000
LDL-C, mmol/L	3.85 (3.08, 4.64)	3.14 (2.54, 3.95)	*Z*=-3.245	0.001
ALT, U/L	27.00 (16.00, 43.00)	25.00 (20.00, 37.25)	*Z*=-0.022	0.983
AST, U/L	23.00 (18.00, 31.00)	27.00 (19.00, 39.25)	*Z*=-1.310	0.190
TBIL, μmol/L	13.00 (9.40, 18.70)	12.35 (8.50, 18.88)	*Z*=-0.051	0.959
ALP, U/L	81.00 (65.00, 98.00)	76.50 (62.00, 105.25)	*Z*=-0.198	0.843
GGT, U/L	36.00 (21.00, 69.00)	29.00 (16.75, 70.75)	*Z*=-0.375	0.708
LDH, U/L	218.00 (180.00, 294.00)	280.00 (194.25, 368.00)	*Z*=-2.587	0.010
Albumin, g/L	45.70 (42.20, 48.30)	43.40 (38.45, 49.05)	*Z*=-1.429	0.153
Cr, μmol/L	62.00 (52.00, 74.00)	61.50 (49.25, 80.75)	*Z*=-0.764	0.445
BUN, mmol/L	4.50 (3.70, 5.10)	5.05 (3.50, 5.93)	*Z*=-1.928	0.054
Glucose, mmol/L	10.30 (7.80, 16.90)	15.85 (13.45, 20.58)	*Z*=-3.170	0.002
Ca^2+^, mmol/L	2.33 (2.18, 2.40)	2.24 (1.84, 2.37)	*Z*=-2.165	0.030
D-D, ug/mL	0.46 (0.25, 1.07)	1.57 (0.52, 4.23)	*Z*=-3.797	0.000
Ascites, n (%)	19 (11.6)	15 (60.0)	*χ*^2^ = 31.262	0.000
BISAP score	1.0 (0.0,1.0)	2.0 (1.0, 2.0)	*Z*=-5.856	0.000

HTG-NSAP, hypertriglyceridemic non-severe acute pancreatitis; HTG-SAP, hypertriglyceridemic severe acute pancreatitis; WBC, white blood cell count; NEU, neutrophil count; HCT, haematocrit; RBC, red blood cell count; PLT, platelet count; Hb, haemoglobin; CRP, C-reactive protein; SAMY, serum amylase; TG, triglyceride; TCHOL, total cholesterol; HDL-C, high-density lipoprotein cholesterol; LDL-C, low-density lipoprotein cholesterol; ALT, alanine aminotransferase; AST, aspartate aminotransferase; TBIL, total bilirubin; ALP, alkaline phosphatase; GGT, Gamma-glutamyl transferase; LDH, lactate dehydrogenase; Cr, creatinine; BUN, blood urea nitrogen; Ca^2+^, serum calcium; D-D, D-dimer; BISAP, Bedside Index for Severity in Acute Pancreatitis.

### 3.3 Multivariate logistic regression analysis of HTG-SAP

Parameters showing significant differences between groups in univariate analysis (*P* < 0.05) included WBC, NEU, CRP, SAMY, TCHOL, HDL-C, LDL-C, LDH, glucose, Ca^2+^, D-D, the presence or absence of ascites, and BISAP score. They were advanced to subsequent multivariate logistic regression modeling. Multivariate analysis revealed that BISAP score, LDL-C level, and the presence or absence of ascites served as independent predictors of HTG-SAP (**Table 3**). Among them, the BISAP score (*P* = 0.005, OR = 3.771, 95% CI: 1.503-9.463), the presence of ascites (*P* = 0.006, OR = 7.276, 95% CI: 1.781-29.717), and low LDL-C (*P* = 0.004, OR = 0.338, 95% CI: 0.161-0.709) were independent risk factors.

**Table 3:** Multivariate logistic regression analysis of independent predictors of HTG-SAP.

Variables	B	OR	95% CI	*P* value
LDL-C	-1.085	0.338	0.161-0.709	0.004
Ascites	1.985	7.276	1.781-29.717	0.006
BISAP score	1.327	3.771	1.503-9.463	0.005
Constant	0.348	1.416	-	0.917

HTG-SAP, hypertriglyceridemic severe acute pancreatitis; LDL-C, low-density lipoprotein cholesterol; BISAP, Bedside Index for Severity in Acute Pancreatitis; OR, odds ratio; CI, confidence interval.

### 3.4 Diagnostic value of independent predictors and the combined predictive model for HTG-SAP

**Figure 1:**
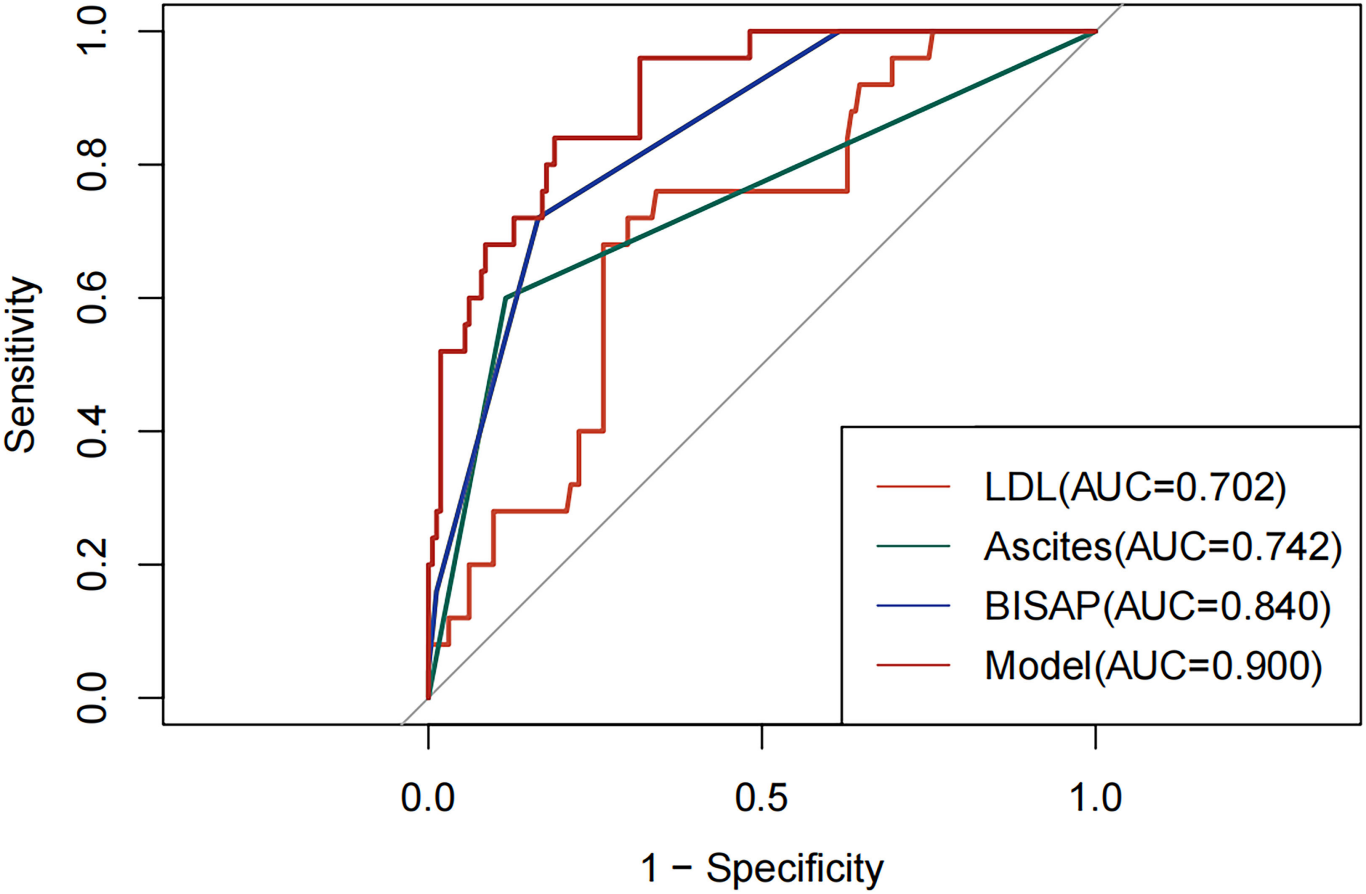
Receiver operator characteristic curve of independent predictors and predictive model for hypertriglyceridemic severe acute pancreatitis (HTG-SAP) AUC, area under the curve; LDL, low-density lipoprotein cholesterol; BISAP, Bedside Index for Severity in Acute Pancreatitis.

Based on the multivariate analysis results, a logistic regression model was developed: Logit (P)=0.348+1.327×BISAP score +1.985×Ascites −1.085×LDL−C. The combined BISAP score, ascites, and LDL-C model for predicting the severity of HTG-AP was established. The predictive performance quantified by ROC analysis yielded AUC values of 0.840 for BISAP score, 0.742 for ascites, and 0.702 for LDL-C. The combined AUC for BISAP score, ascites, and LDL-C was 0.900, which was superior to those obtained for BISAP score, ascites, and LDL-C (**Figure 1**). The sensitivity, specificity, and the maximum Youden index were calculated for these parameters and the novel model, with optimized cutoff values determined as 1.50, 0.50, 3.29, and 0.11, respectively (**Table 4**).

**Table 4:** Diagnostic value of LDL-C, Ascites, BISAP score and predictive model in HTG-SAP.

Variables	AUC	Cut-off	Sensitivity	Specificity	Youden index	95% CI
LDL-C	0.702	3.29	0.702	0.701	0.403	0.602-0.802
Ascites	0.742	0.50	0.600	0.884	0.484	0.622-0.862
BISAP score	0.840	1.50	0.720	0.835	0.555	0.767-0.914
Predictive model	0.900	0.11	0.840	0.811	0.651	0.842-0.957

LDL-C, low-density lipoprotein cholesterol; BISAP, Bedside Index for Severity in Acute Pancreatitis; HTG-SAP, hypertriglyceridemic severe acute pancreatitis; AUC, area under the curve; CI, confidence interval.

### 3.5 Assessing the clinical utility of the model

**Figure 2:**
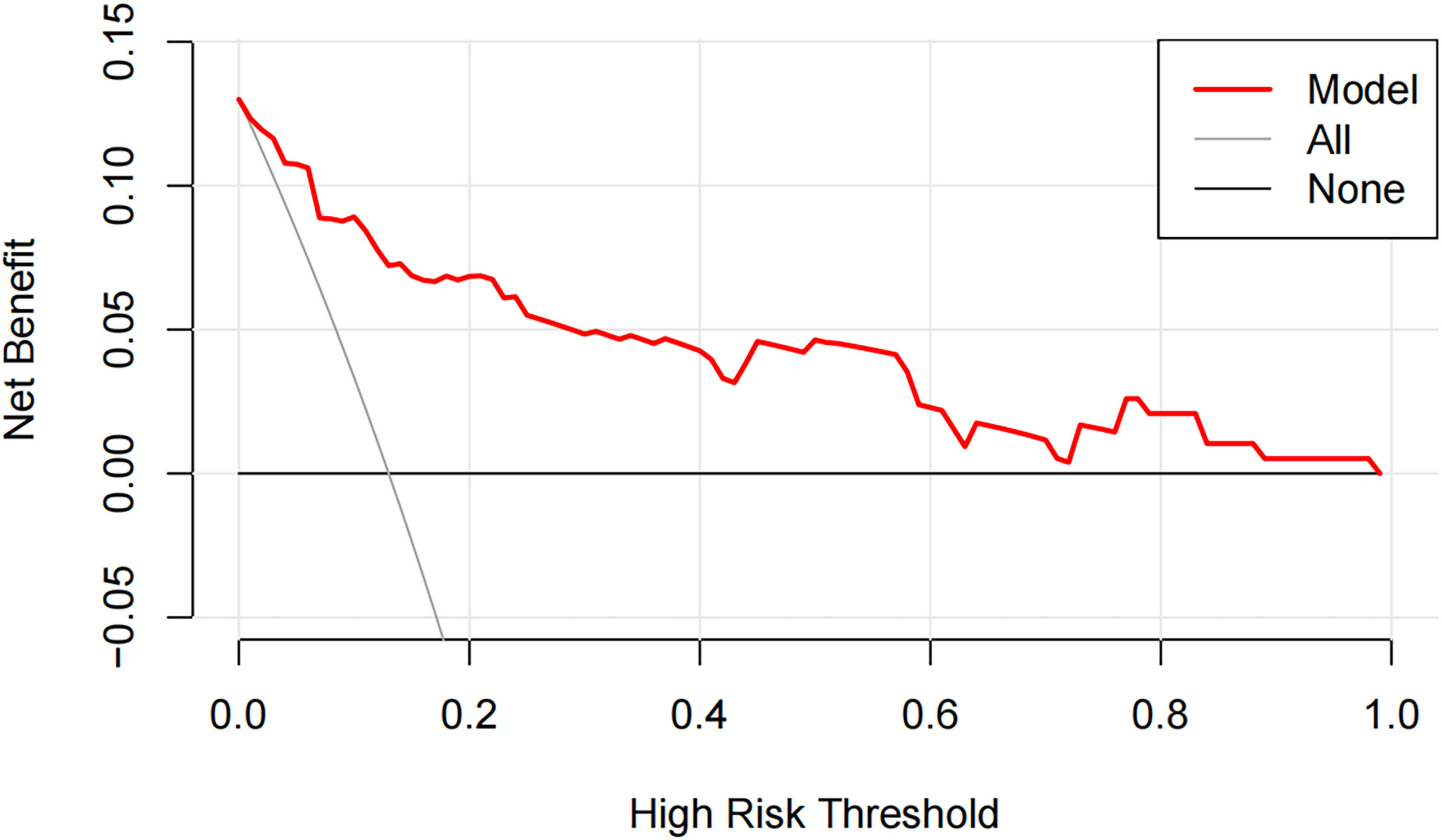
Decision curve analysis for the model in predicting hypertriglyceridemic severe acute pancreatitis (HTG-SAP) The Y-axis represents the net benefit. The X-axis represents the threshold probability. The red solid line depicts the net benefit of the novel prediction model for HTG-SAP. The gray solid line represents the strategy of intervening for all patients, and the black solid line represents the strategy of intervening for none.

DCA was employed to evaluate whether using the novel prediction model to guide clinical decision-making could provide net benefit. The results demonstrated that the predictive model curve remained above both reference lines (all and none) at threshold probabilities greater than 0, indicating that timely and effective clinical interventions triggered by positive predictions of HTG-SAP using our model would yield substantial clinical benefits. (**Figure 2**).

### 3.6 Internal validation of the model

**Figure 3:**
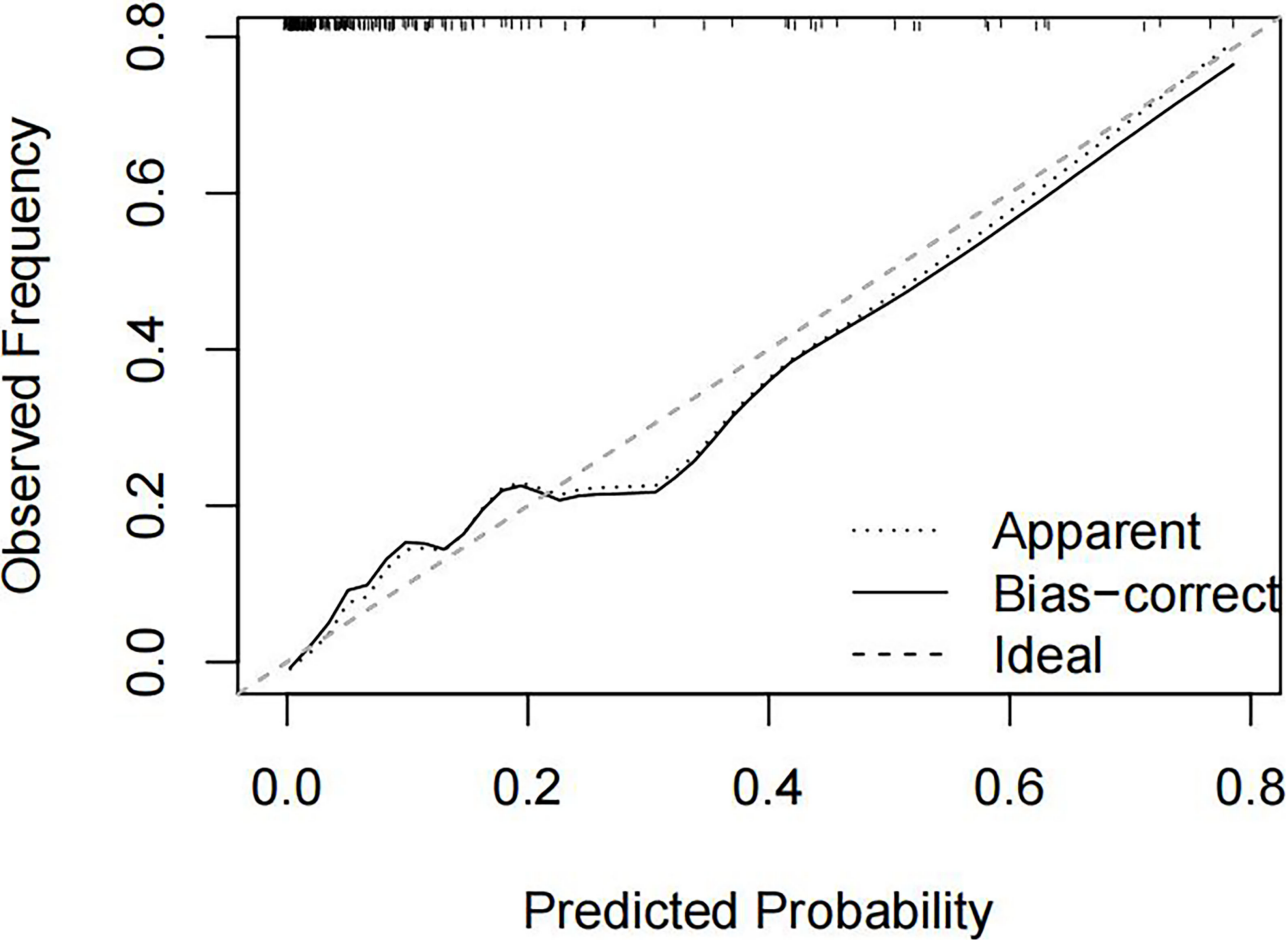
Calibration curve of hypertriglyceridemic severe acute pancreatitis (HTG-SAP) model The X-axis and Y-axis show the predicted and actual probabilities of HTG-SAP, respectively. A perfect prediction would align with the 45-degree diagonal (dashed ideal line). The apparent calibration curve (black dashed line) represents performance on the original data, whereas the bias-corrected curve (black solid line) shows the performance following adjustment for optimism using 1000 bootstrap resamples.

We evaluated the calibration of the model using 1000 bootstrap resamples. The bootstrap-corrected calibration curve demonstrated reasonable agreement between predicted probabilities and observed outcomes (**Figure 3**).

## 4 Discussion

As the incidence of HTG-AP continues to rise recently (7, 38), with significant morbidity and mortality in severe cases, early recognition of patients at risk for HTG-SAP is critically important, especially within 24 hours of admission. Owing to increasing research on HTG-AP, the epidemiology, clinical characteristics, and treatment measures of HTG-AP have been identified (39); however, prediction is challenging and more reliable prediction models are needed. The precise pathophysiology mechanisms underlying the onset and progression of HTG-AP remain incompletely elucidated to date. Studies have shown that hydrolysis of surplus TG by pancreatic lipase results in free fatty acid deposition, which induces both acinar cell damage and microvascular injury in pancreatic tissue; Concurrently, chylomicrons elevate plasma viscosity, precipitating regional ischemia and impairing microcirculation (40, 41). The pathophysiological distinction of HTG-AP from other AP etiologies suggests potential differentiation in prognostic biomarker patterns. Consequently, this study is designed to evaluate the clinical utility of integrating the BISAP score with complementary parameters for severity stratification in patients with HTG-AP. In our retrospective study, we identified two independent predictors in addition to the BISAP score: LDL-C and the presence of ascites. Furthermore, the combined model had a good clinical predictive effect.

Our study demonstrated that LDL-C levels significantly decreased during the early phase of HTG-SAP. Both TG and cholesterol play crucial roles in lipid homeostasis and are likely intricately involved in the initiation and perpetuation of inflammatory processes (42). Dysregulated cholesterol metabolism may amplify inflammatory responses, which is associated with both the pathogenesis and progression of SAP as well as poor clinical outcomes. Conversely, AP can also disrupt cholesterol metabolism, resulting in reduced serum levels of cholesterol-related lipids such as LDL-C (33). The inflammatory state triggers a reduction in circulating LDL-C levels through multiple pathways. Upregulation of LDL-C receptors enhances cellular uptake and catabolism of LDL-C (43), while CRP binds to LDL-C, forming complexes that are rapidly cleared (44). Furthermore, lower LDL-C levels are also correlated with a higher severity of AP. Pathophysiologically underlying this phenomenon are two principal mechanisms: 1) suppressed hepatic synthesis of LDL-C due to the early release of pro-inflammatory cytokines such as IL-6 and TNF-α (45), and 2) increased capillary permeability, leading to a shift of lipoproteins from the intravascular to the extravascular space (46). Accumulating evidence indicates that decreased serum LDL-C levels following AP onset are significantly associated with disease severity and clinical outcomes. Khan et al. (47) reported lower LDL-C levels within 48 hours of admission in patients with SAP, showing inverse correlation with in-hospital mortality and prolonged hospitalization (r = − 0.264). Similarly, Peng et al. (48) observed significantly reduced LDL-C levels within 24 hours in patients developing persistent OF, which was closely associated with increased mortality risk in cases of SAP. A recent study by Hong et al. revealed an association between LDL-C and the development of SAP (49). Notably, they suggested a U-shaped association between LDL-C levels and SAP risk, demonstrating that both low LDL-C (< 90 mg/dL; OR 3.05) and high LDL-C (> 150 mg/dL; OR 4.42) within 24 hours post-admission were independently associated with increased SAP risk (32). These findings indicate that LDL-C may serve as a potential risk factor or early predictor of SAP, a conclusion that aligns with the findings of our present investigation. Multifactor analysis showed that low LDL-C was an independent risk factor for HTG-SAP. Furthermore, the uniqueness of our study lied in its exclusive focus on AP with HTG as the etiological type. However, our findings also demonstrated that using LDL-C alone for predicting HTG-SAP yielded limited performance, with an AUC of merely 0.702 and a sensitivity of only 70.2%. Therefore, LDL-C alone may no longer offer an advantage, and combining several clinical parameters or integrating LDL-C into clinical scoring systems may improve the predictive accuracy of HTG-SAP.

Our study revealed that the presence of ascites within 24 hours of admission was an independently predictive factor with the AUC value of 0.742 and specificity of 88.4%. Ascites in AP are caused by increased capillary permeability, subsequent fluid extravasation, and diminished colloidal osmotic pressure in blood vessels due to albumin consumption and mechanical exudation. Additionally, the development of ascites in early-stage AP is primarily driven by pancreatic parenchymal necrosis and loss of structural integrity in the pancreatic ducts (50). Pancreatic ascites, rich in trypsin, pancreatic lipase, unsaturated fatty acids, and cytokines, introduces these toxic components into the systemic circulation via lymphatic vessels, which are key drivers of MODS and abdominal compartment syndrome in the early stage of SAP (27). The early formation of ascites in AP has been established as both a clinically significant severity marker and an important predictor for local complications, according to previous research (50). Zeng et al. reported that the presence of ascites was a predictor of local and systemic AP complications, with patients exhibiting ascites being at significantly higher risk of progressing to severe disease and developing adverse clinical outcomes (51). Emerging evidence indicates that AP patients presenting with ascites exhibited substantially increased incidence of OF, severity scores, IPN, and mortality (27, 52). A retrospective study by Shuanglian et al. (28) reported that the presence of ascites served as an independent predictor for HTG-SAP, a finding consistent with the conclusions of our present study. Furthermore, ascites is one of the extrapancreatic complications in MCTSI. CTSI or MCTSI provides reliable radiological assessment of pancreatic inflammatory changes and necrotic areas, facilitating stratification of AP severity in clinical practice. A meta-analysis showed that CTSI or MCTSI was a good predictor of both mortality and AP severity, especially considering severity of AP, they were revealed as equally valuable as BISAP, APACHE II, or Ranson score (53). However, the most recent guidelines of AP recommend that enhanced CT is performed 48–72 hours after the onset of the symptoms, which is unsuitable for early AP prediction (3). CT of the abdomen is usually needed to confirm the diagnosis and identify the cause at admission. Ascites, as abdominal imaging finding, was integrated into the combined model, which could improve the discrimination accuracy and measurement reliability.

The BISAP score is designed for bedside assessment of AP severity within 24 hours of hospital admission, which incorporates five clinical parameters worth one point each: BUN > 25 mg/dL, impaired mental status (Glasgow Coma Scale, GCS < 15), presence of SIRS, age > 60 years, and pleural effusion. The stratification capacity of the BISAP scoring system effectively delineates patients along a risk continuum, where ascending scores correspond to increasing probabilities of SAP and fatal outcomes (19). In a prospective study of 50 patients with AP, the BISAP score (with cutoff value ≥ 2) achieved 84% predictive accuracy for SAP, showing comparable performance to the APACHE-II (54). In a prospective observational study, the BISAP score demonstrated significant predictive accuracy for severity stratification, organ dysfunction development, and fatal outcomes in AP, as good as APACHE-II, but outperforming the Ranson score and CTSI (55). A retrospective analysis carried out on 108 patients with acute biliary pancreatitis showed the BISAP score (AUC 0.91), CRP levels at 48 hours (AUC 0.92), MCTSI (AUC 0.94), and CTSI (AUC 0.93) had the highest AUC for predicting the severity of AP (35). Recent study showed the BISAP score performed better than APACHE II and the sequential organ failure assessment score, and was superior to other predictors in the prediction of SAP during the first 24 hours (36). Our study only analysed data of patients with HTG-AP and showed the BISAP score demonstrated good predictive performance for HTG-SAP during the first 24 hours, with the AUC of 0.840, which corroborate previous studies. However, Wu et al. analysed 1,848 AP cases and found that the sensitivity of BISAP to predict SAP was only 64.9% (26). Another meta-analysis involving 1,972 subjects of AP reported that BISAP demonstrated low sensitivity (64.82%), highlighting its limitations as an independent severity assessment tool (25). This study revealed the sensitivity of the BISAP score was 72.0%, which is inconsistent with previous reports. It may stem from the distinctive pathogenic mechanisms underlying HTG-AP or deviations resulting from the limited number of severe cases available for analysis. Future multicenter studies with larger cohorts are required to validate these findings and further elucidate the value of the BISAP score in assessing HTG-SAP.

Previous studies have combined scoring models or clinical biochemical indicators and yield superior predictive performance compared to a single prognostic score or individual markers. Ratiu et al. (35) found combination of the BISAP score, CTSI, and CRP level at 48 h could increase the accuracy of predicting severity in patients with acute biliary pancreatitis. But it is not suitable for early admission assessment. Lu et al. (34) reported BISAP score combined with CRP and NLR had a higher predictive value for early severity assessment of AP. However, these studies all lack tools for the early severity prediction of HTG-AP. Shuanglian et al. (28) proposed a prediction model comprising CRP, LDH, Ca^2+^, and ascites, which demonstrated high accuracy and sensitivity in predicting HTG-SAP. However, their study included only 42 severe cases and large-sample, multicenter studies are warranted to validate these findings. Our study developed a novel model by integrating the classic BISAP score with LDL-C and the imaging indicator of ascites. This model demonstrated not only high accuracy (AUC 0.900, 95% CI 0.842-0.957) for early prediction of HTG-AP severity, but also exhibited high sensitivity (84.0%) and specificity (81.1%). Furthermore, the level of LDL-C and abdominal CT are routinely available and easily obtained, and their incorporation into the BISAP score may compensate for its limitations. This study suggested that HTG-AP patients with BISAP score ≥ 2, LDL-C < 3.29 mmol/L, and the presence of ascites early after admission might be at potential risk of progressing to HTG-SAP. This should alert clinicians to intensify monitoring and initiate early intervention, thereby potentially improving outcomes and streamlining resource allocation. The DCA also indicated our model is clinically useful. If a physician chooses to intervene for patients with a predicted probability of HTG-SAP above 0%, using our model would lead to better outcomes.

This study had several limitations. Firstly, it was a single-center study with a small sample size. The limited number of severe cases (n = 25) might reduce the statistical power of the multivariate model and imply overfitting. This likely reflects the low population prevalence of HTG-SAP. Additionally, these data lack external validation. Our analysis only indicated that the predictive model might have potential predictive utility. Future studies with expanded sample sizes and multicenter validation are necessary to verify the clinical utility and generalizability of the proposed model. Secondly, as a retrospective single-center investigation with restricted cohort size, this study inherently faced selection bias due to demographic variations in HTG-AP manifestations across geographical regions, ethnic groups, and lifestyle factors. Additionally, retrospective assessment of subjective neurological symptoms (e.g., mental status changes) through medical records introduced potential information bias. A prospective validation of these findings is mandatory prior to clinical application. Thirdly, LDL-C measurement demonstrates potential variability during the acute phase of an illness. The role of LDL-C levels in the development of HTG-AP remains to be clarified. Finally, the underlying mechanisms linking these predictors to the pathogenesis of HTG-AP require further elucidation.

## 5 Conclusion

In conclusion, the BISAP score, low LDL-C level, and ascites were independent risk factors for predicting HTG-SAP in our study. The combination of the BISAP score, LDL-C, and ascites might serve as a potential useful model for evaluating the severity of HTG-AP within 24 hours of admission. Its application could provide early guidance for clinical treatment, thereby helping to mitigate the risk of disease progression to severe forms and reduce mortality. However, it is important to emphasize that our study is an exploratory investigation with a limited sample size. Additional multicenter studies involving larger samples are needed to confirm these results.

## Data Availability

The raw data supporting the conclusions of this article will be made available by the authors, without undue reservation.
